# Polyol-Functionalized Manganese-Based Nanoparticles: From Insulin Amyloid Inhibition to Nanozyme-Mediated Antioxidant Activity

**DOI:** 10.3390/ijms27188321

**Published:** 2026-09-18

**Authors:** Kleoniki Giannousi, Zoi Kourpouanidou, Elpida Pantelidou, Catherine Dendrinou-Samara

**Affiliations:** Laboratory of Inorganic Chemistry, School of Chemistry, Aristotle University of Thessaloniki, 541 24 Thessaloniki, Greece; zkourpo@physics.auth.gr (Z.K.); elpipant@gmail.com (E.P.); samkat@chem.auth.gr (C.D.-S.)

**Keywords:** manganese nanoparticles, insulin amyloidosis, insulin aspart, insulin glargine, thioflavin-T fluorescence, hydrogen peroxide scavenging, nanozymes

## Abstract

Insulin amyloidosis poses challenges in diabetes therapy and biopharmaceuticals handling. Here, functionalized manganese-based nanoparticles (MnCO_3_@TEG, MnCO_3_@OAm, MnOHCO_3_@TEG, MnO_2_/Mn_2_O_3_@TEG, and Mn_3_O_4_@PG) were synthesized via solvothermal routes and evaluated *in vitro* using a fresh insulin aspart formulation (NovoRapid) and a separately aged insulin glargine formulation (Lantus). Structural and colloidal characterization (ATR-FTIR, XRD, TGA, DLS, zeta potential) confirmed phase purity and distinct surface profiles. In the fresh insulin aspart system, all nanoparticles extended the nucleation lag phase, with MnCO_3_@TEG delaying fibrillogenesis from 21.16 h to 105.41 h. In the aged insulin glargine system containing pre-existing ThT-positive aggregates, small, negatively charged nanoparticles (MnOHCO_3_@TEG, Mn_3_O_4_@PG) re-established a distinct lag phase and suppressed further aggregation. In the ThT decay assays, highly dispersed Mn_3_O_4_@PG produced the largest decrease in amyloid-associated fluorescence, to ~40% of the initial signal, whereas large clusters (MnO_2_/Mn_2_O_3_@TEG, 624 nm) produced a smaller decrease; limited access arising from steric effects is one possible explanation. Additionally, structural hydroxyl groups and high colloidal stability enabled MnOHCO_3_@TEG (−38.7 mV) to exhibit superior catalase-like H_2_O_2_ scavenging (42.27% inhibition at 250 μg/mL). Overall, fine-tuning nanoparticle size, surface charge, and core composition may offer a promising dual-action strategy for targeting insulin amyloidosis and associated oxidative stress across different stages of fibril formation, while further structural characterization and biological safety studies are needed to establish its therapeutic relevance.

## 1. Introduction

The misfolding of proteins and peptides into amyloid aggregates has been associated with more than 50 human disorders, including the most common neurodegenerative diseases, Alzheimer’s and Parkinson’s, as well as type 2 diabetes [1]. These aggregates share a common β-sheet-rich conformation, referred to as a cross-β structure, independent of the sequence and topology of the monomeric protein precursor. In this characteristic structure, the monomeric units are stacked one on top of the other, with their β-strands disposed vertically to the elongating axis, generating the typical fibrillar morphology [2]. An important statement is that these prefibrillar oligomers may represent the principal toxic entities in many amyloid diseases, rather than mature fibrils [3]. Focusing on insulin fibrillation, in diabetic patients, subcutaneous insulin injections may cause a series of injection-site complications, like lipohypertrophy, lipoatrophy, and insulin-derived amyloidosis. Insulin-derived amyloidosis is a localized cutaneous amyloidosis characterized by deposits of insulin fibrils at or near the site of injections. This mass is also called an “insulin ball”. These deposits lead to poor glycemic control due to insufficient absorption of insulin and a reduced amount of active monomeric insulin [4,5].

A plethora of additives have been used to stabilize the native or oligomeric states of insulin. These inhibitors interfere with intermediates or remodel pre-formed fibrils into less toxic species. They range from small phenolic molecules, osmolytes, peptides, and nanoparticles, and they act primarily through non-covalent interactions (hydrogen bonds, electrostatic forces, hydrophobic contacts, π-π stacking) with specific regions of the insulin molecule [6]. Despite the utility of non-covalent additives, their translation is bottlenecked by weak binding reversibility, lack of intrinsic catalytic activity, and an inability to counteract reactive oxygen species (ROS)-driven microenvironmental damage. The organic molecules often require high, near-stoichiometric concentrations to be effective, and at these doses, many phenolic compounds exhibit systemic cytotoxicity, poor solubility, or off-target membrane interactions [5,7,8]. Inorganic and metallic nanomaterials, including gold nanoparticles, titanium dioxide (TiO_2_) NPs, and black phosphorus quantum dots, have been widely explored to modulate amyloidosis pathways [9,10,11]. However, their performance in complex biological systems is often compromised by non-specific protein corona formation, which can unpredictably alter aggregation kinetics or even promote fibril nucleation [12,13,14,15]. Furthermore, transition-metal-based inorganic cores frequently undergo dissolution and ion leaching in biological fluids, triggering off-target cytotoxicity or initiating pro-oxidant Fenton-like reactions that generate destructive hydroxyl radicals [11,15]. We have previously reported the synthesis of ZnO nanoflowers and polyol-coated ZnO NPs of relatively small size (40 nm) with cylindrical shape. Both nanomaterials affected the amyloid formation mechanism as well as their disaggregation; ZnO nanoflowers, with their sharp edges, exhibited the greatest amyloid degradation rate in both model proteins (73% and 35%, respectively) and inhibited the most insulin fibril growth, while also restraining the fibrillation process in the case of albumin solution [16]. Moreover, we have demonstrated that MnFe_2_O_4_ nanoparticles functionalized with gallic acid exhibit superior antioxidant and anti-amyloidogenic activities, with enhanced inhibition of insulin fibrillogenesis (74.3%) and depolymerization (78.2%) of amyloid fibrils [17].

Beyond their established utility in cancer theranostics, manganese-based nanoparticles (MnNPs) possess intrinsic properties that make them uniquely suited for modulating protein aggregation pathways and counteracting oxidative stress. Manganese naturally cycles across multiple oxidation states (Mn^2+^, Mn^3+^, and Mn^4+^), endowing Mn-based nanostructures with robust, multivalent redox activity and versatile nanozyme-mimicking capabilities—including catalase- and superoxide dismutase-like catalytic routines [18,19]. Unlike inert noble metals or iron-based nanoparticles that risk generating destructive hydroxyl radicals via Fenton chemistry, manganese centers can safely catalyze the decomposition of ROS such as hydrogen peroxide (H_2_O_2_) and superoxide anions into molecular oxygen and water, thereby maintaining biological microenvironmental homeostasis [18,20]. Crucially, the high specific surface area and tunable surface chemistry of manganese nanostructures provide a multivalent platform to interface directly with protein backbones. When appropriately functionalized, these manganese platforms can sterically interfere with hydrophobic contacts and hydrogen-bonding networks and may influence protein aggregation pathways; the magnitude and direction of the effect depend on composition, coating, and assay conditions [21]. These physicochemical features motivate evaluation of manganese-based nanoparticles in protein aggregation assays. However, biocompatibility is composition-, coating-, dose-, and exposure-dependent and must be established experimentally for each formulation before any biomedical application is proposed.

Herein, the solvothermal synthesis of five functionalized manganese-based nanoparticle formulations is reported, followed by in vitro evaluation of their effects on insulin amyloid-associated ThT fluorescence and H_2_O_2_ scavenging. Two distinct commercial insulin analogues were used: a fresh insulin aspart formulation (NovoRapid) and a separately aged insulin glargine formulation (Lantus). Because the analogues and their excipient systems differ, comparisons were made within each formulation; differences between the two systems cannot be attributed to aging alone. Manganese precursor compounds of different composition, chemical character, and oxidation state were used. More specifically, inorganic salts and manganese complexes such as KMnO_4_, MnCl_2_·4H_2_O, Mn(NO_3_)_2_·xH_2_O, Mn(acac)_2_, and Mn(acac)_3_ were applied. Additionally, two polyols of different molecular weight, propylene glycol and tetraethylene glycol, were used, while the effect of additional agents, like NaOH and oleylamine, which acted as reducing and surfactant/coating agents, was also studied. Through this approach, coated manganese nanoparticles MnCO_3_, MnOHCO_3_, MnO_2_/Mn_2_O_3_, and Mn_3_O_4_ were isolated. The characterization of the NPs was carried out using a combination of techniques, such as infrared spectroscopy (ATR-FTIR), X-ray diffraction (XRD), thermogravimetric analysis (TGA), dynamic light scattering (DLS), and zeta potential measurements, in order to determine the chemical groups on the surface, the thermal behavior, the hydrodynamic size, and the colloidal stability of the nanoparticles. For the in vitro study of insulin amyloidosis, the ThT assay was used, while the nanoparticles were further examined for their antioxidant activity (H_2_O_2_ assay).

## 2. Results

### 2.1. Synthesis and Characterization of Functionalized Manganese-Based Nanoparticles

The manganese-based nanoparticles (S1–S5) were synthesized via a polyol-assisted solvothermal route utilizing a high-pressure stainless steel autoclave. Different manganese precursors were dissolved in liquid polyols, which served as both high-boiling solvents and capping/reducing agents. Where specified, reducing agents were incorporated to modulate nucleation kinetics and particle growth (Table 1).

**Table 1:** Synthetic parameters, precursor formulations, and reaction yields for the polyol-assisted solvothermal synthesis of manganese-based nanoparticles (S1–S5).

Sample	Precursor	Polyol	Reducing Agent	Reaction Yield	Isolated NPs
S1	ΚMnO_4_	TEG	-	66.10%	MnCO_3_@TEG
S2	MnCl_2_∙4H_2_O	PG	OAm	34.05%	MnCO_3_@OAm
S3	Mn(acac)_2_	TEG	NaOH	68.85%	MnOHCO_3_@TEG
S4	Mn(NO_3)2_∙xH_2_O	TEG	-	21.80%	MnO_2_/Mn_2_O_3_@TEG
S5	Mn(acac)_3_	PG	NaOH	38.55%	Mn_3_O_4_@PG

**Figure 1:**
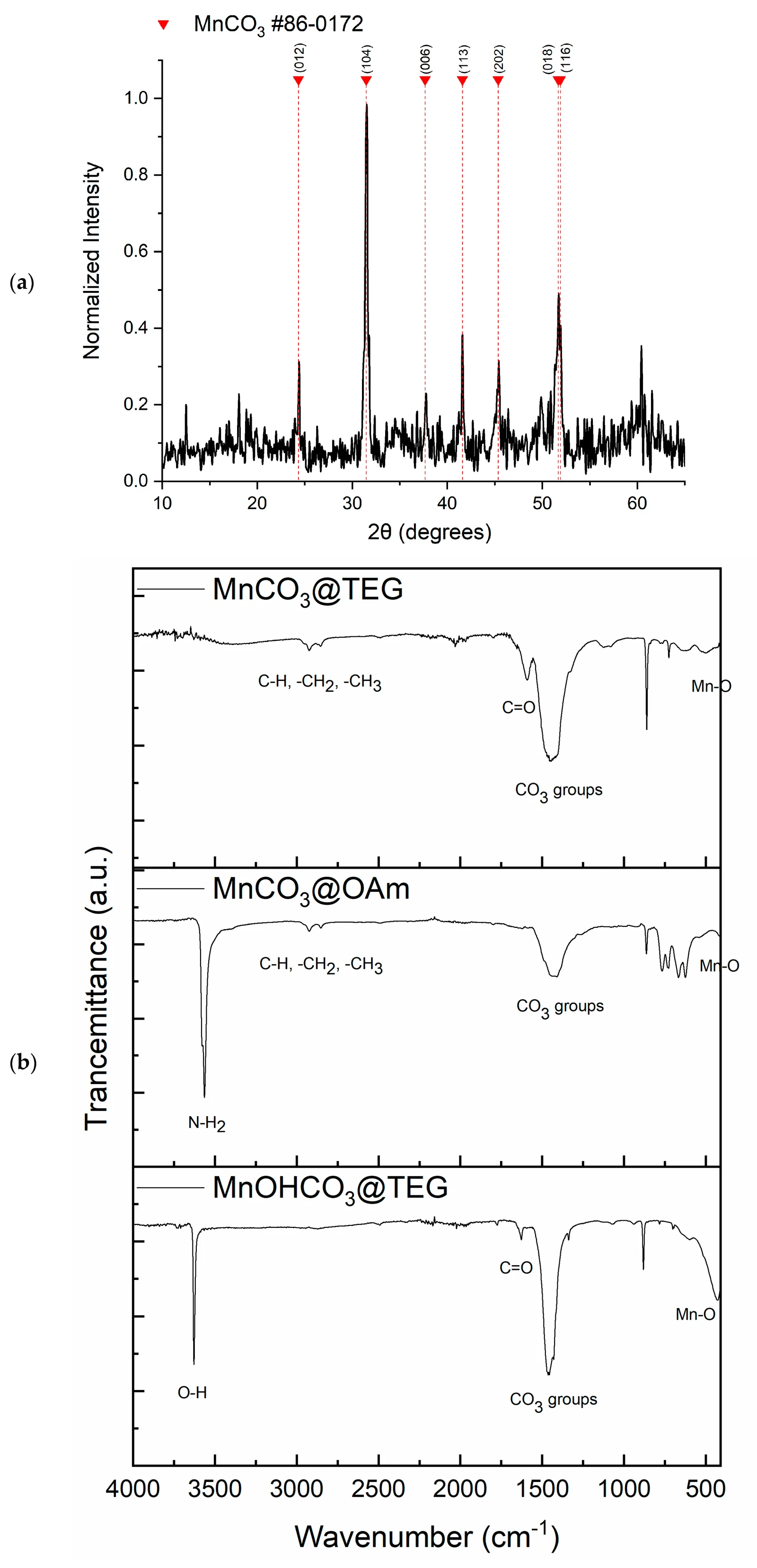

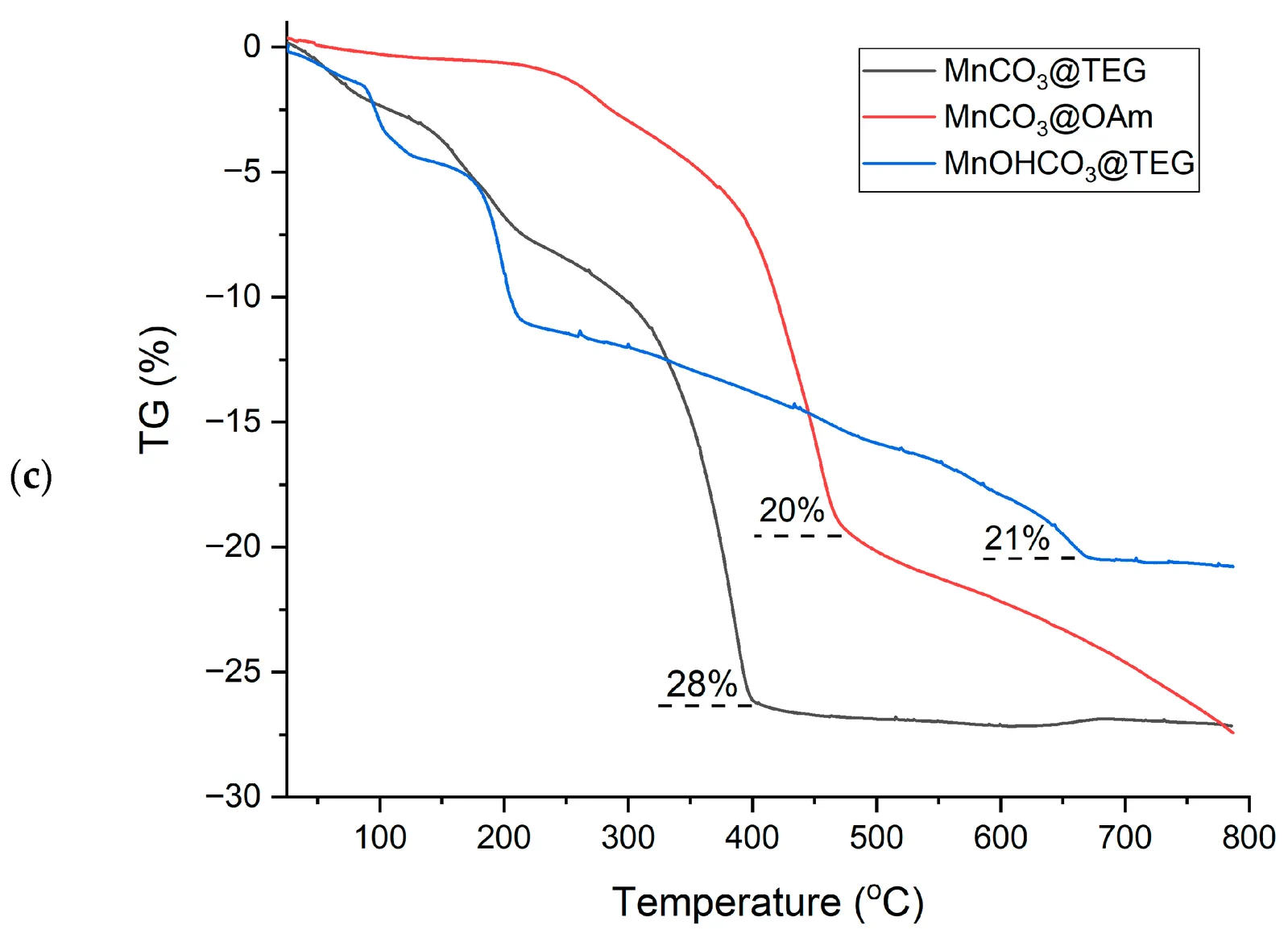
X-ray diffraction pattern of sample S1 (**a**), ATR-FTIR spectra displayed as transmittance (**b**), and thermogravimetric analysis under argon (**c**) of MnCO_3_@TEG, MnCO_3_@OAm, and MnOHCO_3_@TEG.

The identification of the crystalline structure of the three samples, MnCO_3_@TEG, MnCO_3_@OAm, and MnOHCO_3_@TEG, was carried out following the analysis of their XRD patterns. Owing to their analogous diffraction profiles, the XRD pattern of sample S1 is presented as a representative example (Figure 1a). The pattern exhibits well-defined diffraction peaks that match standard ICDD card #86-0172 for pure manganese carbonate (rhodochrosite), confirming it as the sole crystalline phase present. The primary reflections of the structure are indexed to the (012), (104), (006), (113), (202), (018), and (116) planes, with the (104) plane displaying the highest relative intensity. Sample S1 crystallizes in a hexagonal lattice system within the space group R-3c. The calculated lattice parameters for the hexagonal unit cell are α = b = 4.773 Å and c = 15.642 Å, with axial angles of α = β = 90° and γ = 120°.

The ATR-IR spectra (Figure 1b) of all three samples, MnCO_3_@TEG, MnCO_3_@OAm, MnOHCO_3_@TEG, confirm the successful formation of manganese carbonate or hydroxycarbonate cores alongside surface functionalization by their respective organic capping agents. Across all three samples, the presence of the inorganic carbonate core is evidenced by strong characteristic CO_3_^2−^ vibrational bands (~1408–1455 cm^−1^, ~860–880 cm^−1^, and ~702–726 cm^−1^) [22,23], accompanied by low-wavenumber Mn-O stretching modes occupying tetrahedral (~597–665 cm^−1^) and octahedral sites (~430–500 cm^−1^) [24]. The presence of organic shells is identified by aliphatic C-H stretching modes (~2850–2925 cm^−1^) present in all samples [25], along with ligand-specific features: an intense doublet corresponding to N-H_2_ stretching (3579 and 3564 cm^−1^) confirms oleylamine functionalization in MnCO_3_@OAm [26], a sharp structural O-H stretching band (3627 cm^−1^) characterizes MnOHCO_3_@TEG, and polyether C-O-C modes (1123 and 1081 cm^−1^) appear in MnCO_3_@TEG [27]. Furthermore, oxidation products of tetraethylene glycol (TEG) are evident in both TEG-functionalized samples through C=O stretching vibrations (1590 cm^−1^ for aldehyde derivatives in MnCO_3_@TEG and 1626 cm^−1^ for carboxylic species in MnOHCO_3_@TEG) [28].

The thermogravimetric analysis (TGA) of all three samples, MnCO_3_@TEG, MnCO_3_@OAm, and MnOHCO_3_@TEG (Figure 1c), reveals multi-step mass-loss profiles reflecting the sequential evaporation of adsorbed water, degradation of the organic surface coatings, and thermal decomposition of the inorganic core phases. At lower temperatures, each sample exhibits an initial minor mass loss due to the removal of physically adsorbed moisture (~1.5–3.2%), followed by a secondary step corresponding to the breakdown of the organic shell (~3.5–6.5%). The major decomposition stage occurs at higher temperatures and is governed by core breakdown; for MnCO_3_@TEG and MnCO_3_@OAm, the sharp mass decrease between 220–400 °C and 360–500 °C, respectively, corresponds to the decarbonation of the MnCO_3_ core into manganese oxide via CO_2_ evolution. In contrast, MnOHCO_3_@TEG displays an extended degradation region between 250 °C and 680 °C due to two overlapping processes: crystal lattice dehydroxylation and carbonate breakdown. Ultimately, each thermal profile reaches a stable plateau corresponding to the formation of an inorganic manganese oxide residue, yielding total overall mass losses of 28% for MnCO_3_@TEG, 20% for MnCO_3_@TEG MnCO_3_@OAm, and 21% for MnOHCO_3_@TEG, which confirms successful surface functionalization across all three samples.

**Figure 2:**
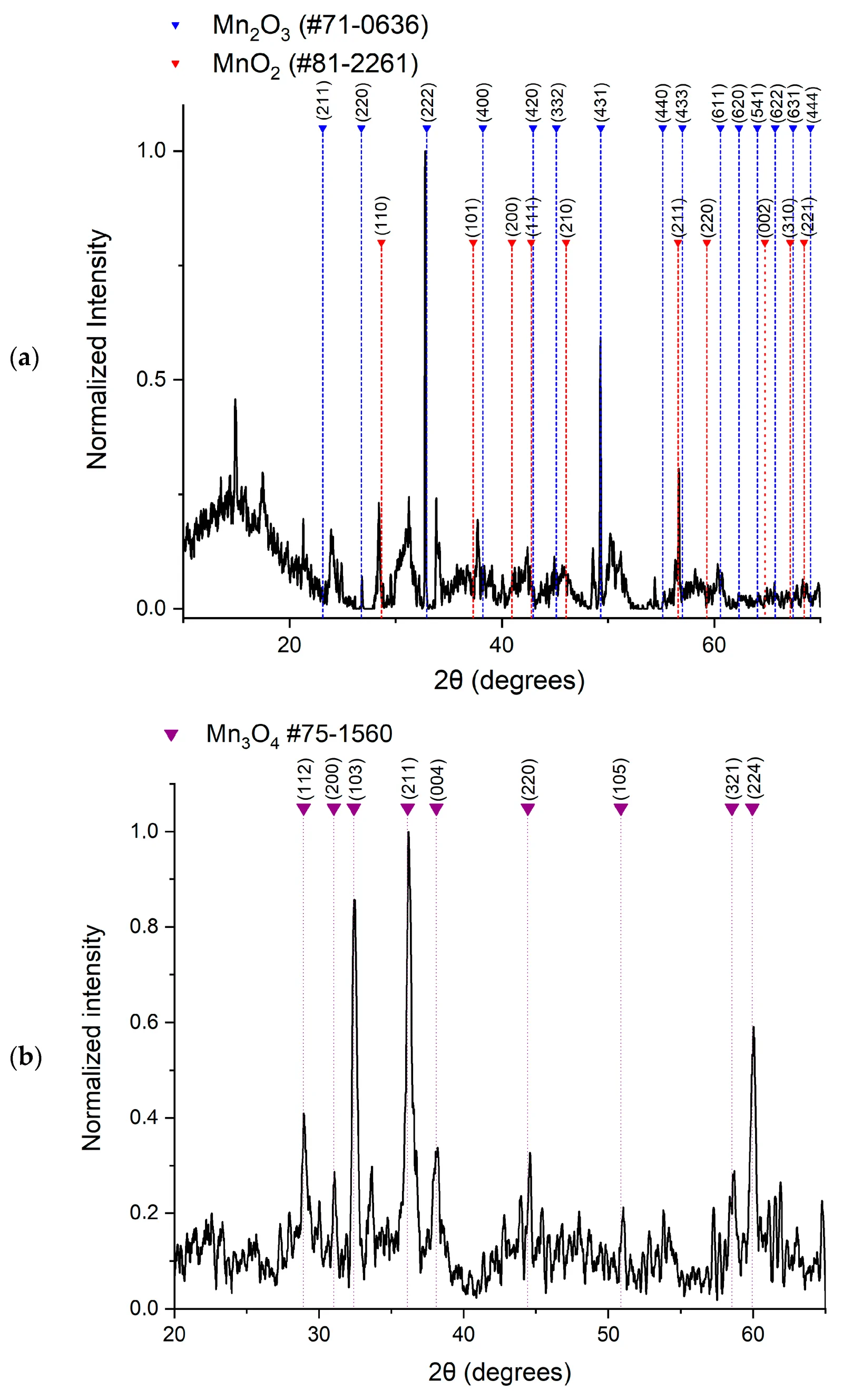

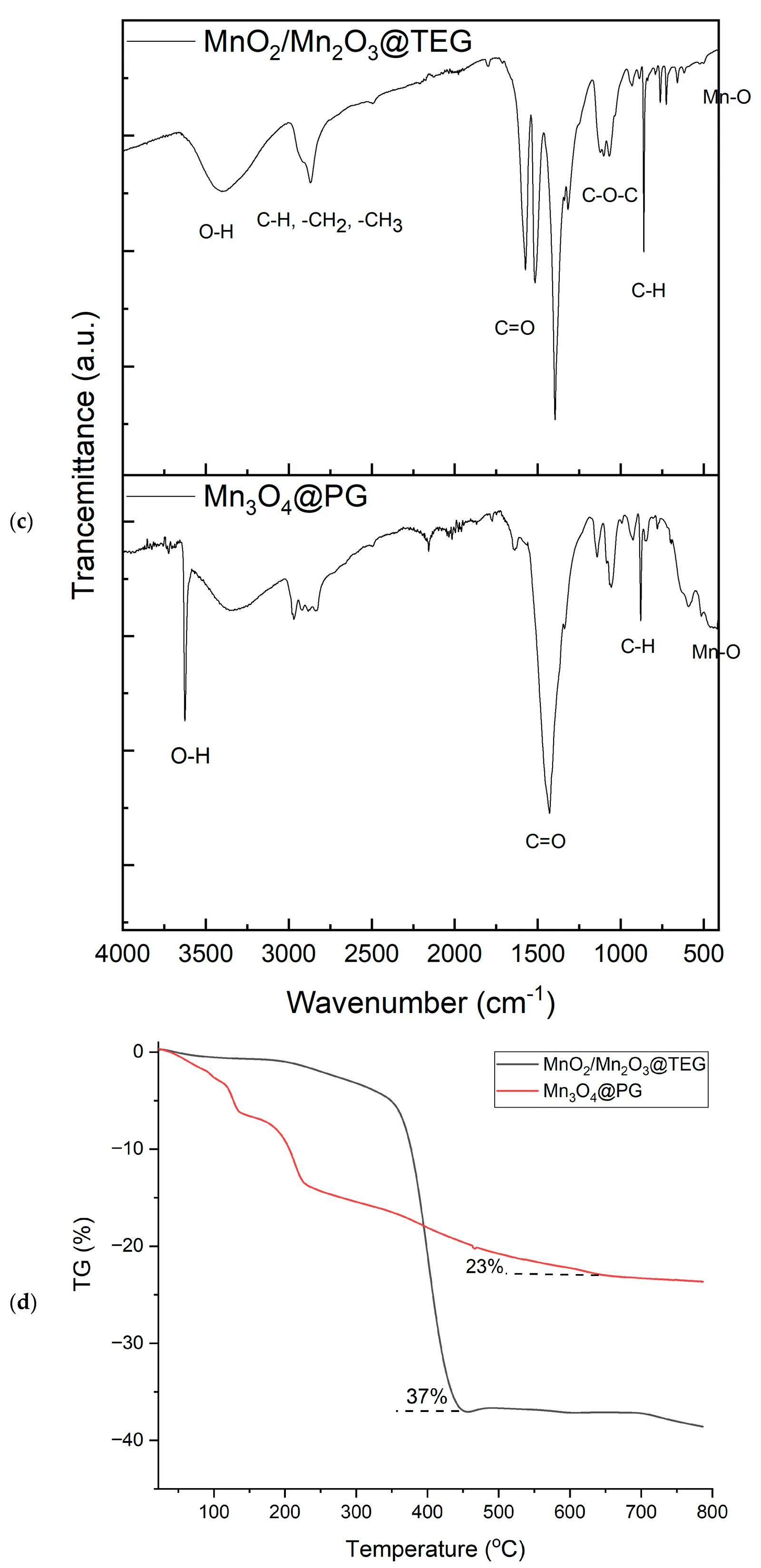
X-ray diffraction pattern of sample S4 (**a**), S5 (**b**), ATR-FTIR spectra displayed as transmittance (**c**), and thermogravimetric analysis under argon (**d**) of MnO_2_/Mn_2_O_3_@TEG NPs and Mn_3_O_4_@PG NPs.

The identification of the crystalline structure of sample S4 was carried out following the analysis of its XRD pattern. The XRD pattern of sample S4 (Figure 2a) exhibits reflection peaks corresponding to ICDD card #71-0636 for bixbyite Mn_2_O_3_ and #81-2261 for pyrolusite MnO_2_. The primary reflections for the bixbyite structure are indexed to the (211), (220), (222), (400), (420), (332), (431), (440), (433), (611), (620), (541), (622), (631), and (444) planes, with (222) being the main peak. Bixbyite Mn_2_O_3_ crystallizes in a cubic lattice structure belonging to the space group Ia-3. The calculated lattice parameters for this cubic structure are α = b = c = 9.4146 Å and α = β = γ = 90°, which are in good agreement with the literature values (α = b = c = 9.409 Å, α = β = γ = 90°) [29]. The characteristic reflections of the pyrolusite MnO_2_ structure are assigned to the (110), (101), (200), (111), (210), (211), (220), (002), (310), and (221) planes. Pyrolusite crystallizes in a tetragonal lattice system with calculated lattice constants of α = b = 4.4041 Å, c= 2.8765 Å, and α = β = γ = 90°, which closely align with the previous reference (α = b = 4.400 Å, c= 2.874 Å, α = β = γ = 90°) [30]. The crystallite size was calculated using the Scherrer equation based on the full width at half maximum (FWHM) of the primary (222) reflection, yielding a value of 140 nm. Quantitative phase analysis indicates that the sample consists of 92% bixbyite and 8% pyrolusite.

The XRD pattern of sample S5 (Figure 2b) exhibits reflection peaks corresponding to ICDD card #75-1560 for hausmannite Mn_3_O_4_, which constitutes the sole phase present in the sample. The main structural reflections are indexed to the (112), (200), (103), (211), (004), (220), (105), (321), and (224) planes, with the (211) plane displaying the primary (most intense) peak. Sample S5 crystallizes in a tetragonal lattice structure. The calculated lattice parameters for this tetragonal structure are α = b = 5.762 Å, c = 9.439 Å, and α = β = γ = 90°, which closely agree with literature values (α = b = 5.762 Å, c = 9.46 Å, α = β = γ = 90°) [31]. The crystallite size was calculated as 28 nm.

The ATR-IR spectra (Figure 2c) of MnO_2_/Mn_2_O_3_@TEG NPs and Mn_3_O_4_@PG NPs confirm the formation of distinct manganese oxide core phases along with their corresponding organic shell modifications. In the high-wavenumber region, hydroxyl features appear as a broad O-H stretching band centered at ~3400 cm^−1^ for MnO_2_/Mn_2_O_3_@TEG NPs due to adsorbed water, whereas Mn_3_O_4_@PG NPs exhibit a sharp, intense band at 3626 cm^−1^ attributed to structural/surface O–H groups from propylene glycol or its derivatives. Both spectra display signature organic features resulting from polyol oxidation during synthesis: MnO_2_/Mn_2_O_3_@TEG NPs displays aliphatic C–H stretching (2915, 2868 cm^−1^), oxidation derivative bands (1574, 1515, 1394, 1316 cm^−1^), ether C–O–C modes (1120, 1065 cm^−1^), and in-plane C–H bending (861, 761 cm^−1^), while Mn_3_O_4_@PG NPs are dominated by a strong C=O stretching vibration at 1430 cm^−1^ (from hydroxyacetone and lactaldehyde derivatives) along with a C–H bending mode at 879 cm^−1^ [32]. Finally, the low-wavenumber lattice region clearly distinguishes the two oxide phases: MnO_2_/Mn_2_O_3_@TEG exhibits characteristic Mn–O stretching bands at 523 and 500 cm^−1^ [33,34], whereas Mn_3_O_4_@PG shows modes at 592 and 472 cm^−1^, corresponding to Mn–O vibrations in tetrahedral and octahedral coordination sites, respectively [35,36].

Thermogravimetric analysis (TGA) up to 800 °C reveals distinct thermal degradation profiles for MnO_2_/Mn_2_O_3_@TEG NPs and Mn_3_O_4_@PG NPs (Figure 2d), reflecting variations in their surface organic coatings. For Mn_3_O_4_@PG NPs, the thermal decomposition proceeds via a multi-step process: an initial 3% mass loss up to 110 °C from physically adsorbed water evaporation, a double-step loss of ~10% between 110 °C and 250 °C due to the volatilization of propylene glycol oxidation derivatives (hydroxyacetone and lactaldehyde), and a final gradual 10% loss up to 800 °C corresponding to the removal of chemisorbed residues, giving an overall mass reduction of 23%. In contrast, MnO_2_/Mn_2_O_3_@TEG NPs exhibit high thermal stability at lower temperatures with a minor water desorption step (~2.5%) up to 250 °C, followed by a single, sharp weight drop of ~34.5% between 250 °C and 450 °C attributed to the complete degradation and combustion of the TEG shell. Above 450 °C, the TEG profile stabilizes into a plateau corresponding to the solid MnO_2_/Mn_2_O_3_ inorganic residue, yielding a total weight loss of 37% and confirming efficient surface functionalization for both nanoparticle systems.

Dynamic light scattering measurements provided intensity-weighted Z-average hydrodynamic diameters of all samples, which are presented in Table 2 along with the polydispersity indices and the zeta potential values. Mn_3_O_4_@PG NPs had the smallest Z-average hydrodynamic diameter (101 nm), PDI (0.130), and a zeta potential of −36.93 mV. MnOHCO_3_@TEG had a Z-average diameter of 196 nm, PDI of 0.278, and zeta potential of −38.7 mV. MnCO_3_@TEG had a Z-average diameter of 226 nm, PDI of 0.183, and zeta potential of −24.3 mV. These ensemble measurements describe dispersion behavior and do not directly determine primary particle morphology or size.

MnCO_3_@OAm showed a larger Z-average hydrodynamic diameter (324 nm), PDI of 0.299, and zeta potential of −14.7 mV. MnO_2_/Mn_2_O_3_@TEG had the largest Z-average hydrodynamic diameter (624 nm), PDI of 0.410, and a near-neutral zeta potential (−4.1 mV), consistent with a broad, aggregated dispersion. Because the measurements were intensity-weighted, larger scattering species can strongly influence the reported hydrodynamic diameter.

Crystallite size and hydrodynamic diameter are distinct quantities. The Scherrer crystallite size estimates the coherent X-ray scattering domain and may represent only part of a particle or an aggregate. DLS reports the hydrodynamic diameter of scattering entities in suspension, including the inorganic core, surface coating, and any aggregates. Thus, the 140 nm Scherrer crystallite size and 624 nm DLS diameter for MnO_2_/Mn_2_O_3_@TEG are compatible with aggregation and/or multicrystallite assemblies.

### 2.2. Influence of Manganese-Based Nanoparticles on Fresh Insulin Aspart and Aged Insulin Glargine Formulations

Two complementary assay systems were evaluated: a fresh insulin aspart formulation (NovoRapid) for examining de novo formation of ThT-positive aggregates and an experimentally aged insulin glargine formulation (Lantus) for examining nanoparticle effects in the presence of pre-existing aggregates. Both short-acting and long-acting insulin analogues, including insulin aspart and insulin glargine, have been reported in association with injection-site insulin-derived amyloidosis. Nevertheless, because the two systems contain different insulin analogues and formulation-specific excipients, they were analyzed independently against their respective formulation-matched controls and were not used as a matched comparison of aging alone.

#### 2.2.1. Effect of Manganese-Based Nanoparticles on the Fresh Insulin Aspart Formulation

**Figure 3:**
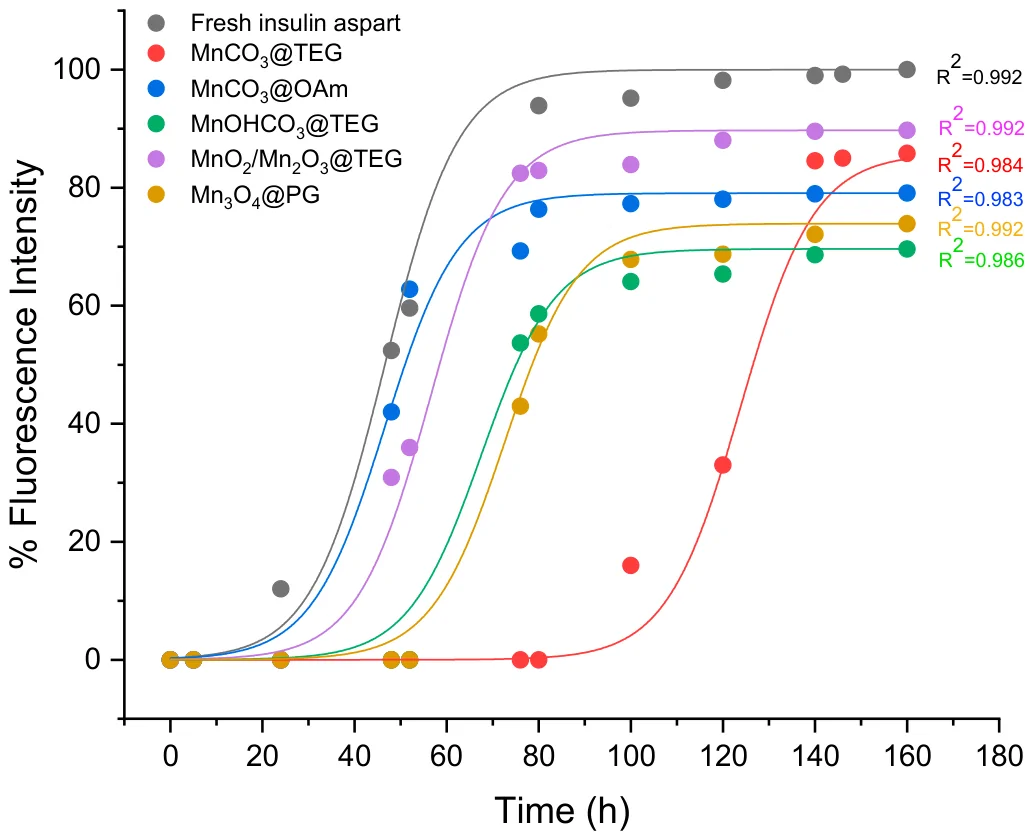
ThT fluorescence profiles of the fresh insulin aspart (NovoRapid) formulation in the absence and presence of manganese-based nanoparticles.

ThT fluorescence profiles for the fresh insulin aspart formulation were monitored in the absence and presence of manganese-based nanoparticles (Figure 3). The control showed a sigmoidal increase in fluorescence. Boltzmann fits indicated rightward shifts and lower terminal fluorescence for nanoparticle-containing samples, and MnCO3@TEG produced the longest fitted lag time. Nanoparticle-only controls containing ThT but no insulin produced no detectable fluorescence under the same measurement conditions, excluding intrinsic nanoparticle emission as the source of the observed signals. Nevertheless, these controls do not exclude nanoparticle-dependent scattering, quenching, displacement of bound ThT, or altered accessibility of ThT-binding sites in samples containing insulin aggregates. The fluorescence profiles, therefore, indicate delayed formation or reduced abundance of ThT-binding species but do not establish fibril mass or morphology.

The kinetic parameters, the elongation rate constant (*k_app_*), the lag time (*t_lag_*), the half time $(t_{1/2}$), and the % aggregation values are given in Table 3. The fitted lag times were 21.16, 105.41, 41.35, 60.42, 36.49, and 63.95 h for the fresh insulin aspart control and the MnCO_3_@TEG, MnCO_3_@OAm, MnOHCO_3_@TEG, MnO_2_/Mn_2_O_3_@TEG, and Mn_3_O_4_@PG nanoparticles, respectively. The corresponding *k_app_* values were 0.081, 0.112, 0.331, 0.214, 0.113, and 0.203 h^−1^. Relative terminal ThT signals were 85.8%, 79.08%, 69.61%, 89.71%, and 73.91% for the five nanoparticle-containing samples. MnOHCO_3_@TEG, therefore, produced the largest reduction in terminal ThT fluorescence under these assay conditions.

**Figure 4:**
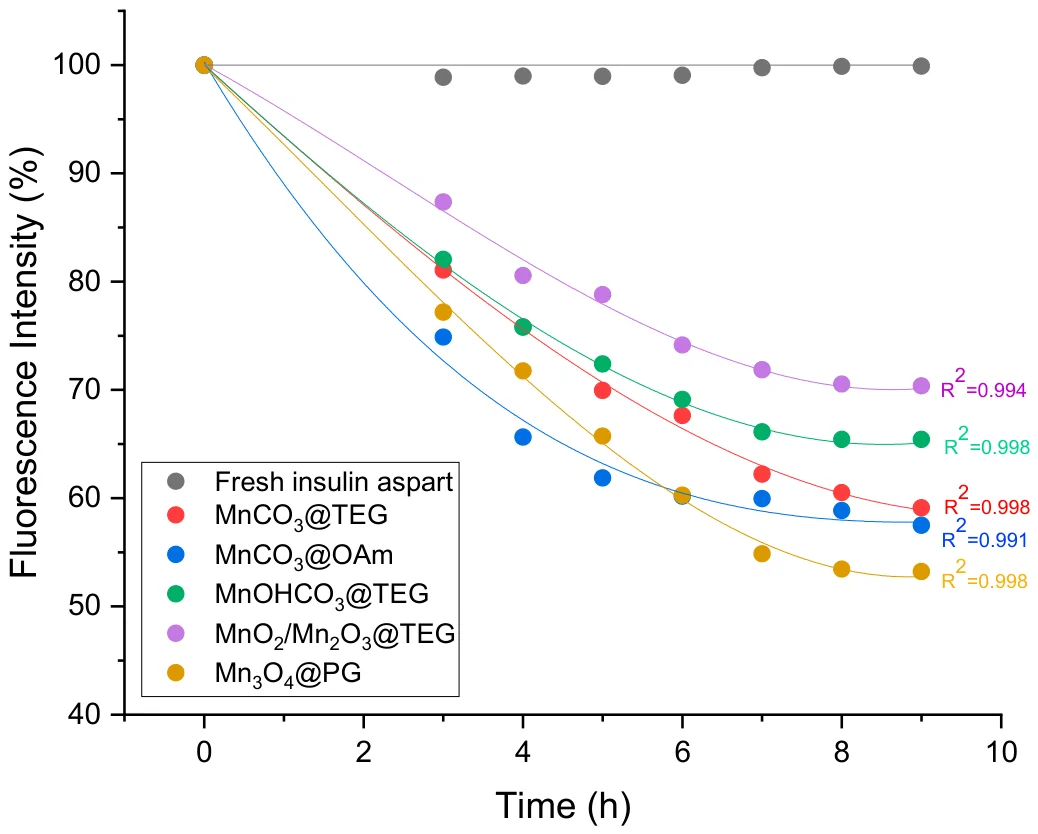
Kinetics of depolymerization of the fresh insulin aspart formulation in the absence/presence of manganese-based nanoparticles.

Changes in ThT fluorescence from pre-formed fresh insulin aspart aggregates after 140 h of incubation were also investigated (Figure 4), as well as the calculated apparent ThT decay parameters, which were based on a third-degree polynomial (cubic) function (Equation (4)) to ensure the highest quality of fit across the complex fluorescence decay profiles, as shown in Table 4. Once the amyloid-associated ThT fluorescence had reached its terminal value, this was designated as time zero. The addition of nanoparticles was then studied for 9 h. The insulin fibril control shows no measurable kinetic constants and retains 100% relative ThT signal. In contrast, all nanoparticle formulations decreased both the apparent half-life and the terminal relative ThT signal. The Mn_3_O_4_@PG nanoparticles (yellow) produced the largest decrease in fluorescence, reaching 53% of the control signal. The MnCO_3_@OAm nanoparticles (blue) display the fastest apparent fluorescence decay kinetics, exhibiting the shortest half-life (2.16 h) and the highest apparent rate constant (0.320 h^−1^). However, they ultimately leave a slightly higher relative ThT signal (57%). The MnCO_3_@TEG (red) and MnOHCO_3_@TEG (green) nanoparticles act more moderately, with half-lives of 3.27 h and 2.85 h, resulting in terminal relative ThT signals of 59% and 65%, respectively. Finally, the MnO_2_/Mn_2_O_3_@TEG nanoparticles (purple) produce the smallest fluorescence decrease relative to the other samples, displaying the longest half-life (3.32 h) and the highest terminal relative ThT signal (70%).

#### 2.2.2. Effect of Manganese-Based Nanoparticles on the Aged Insulin Glargine Formulation

**Figure 5:**
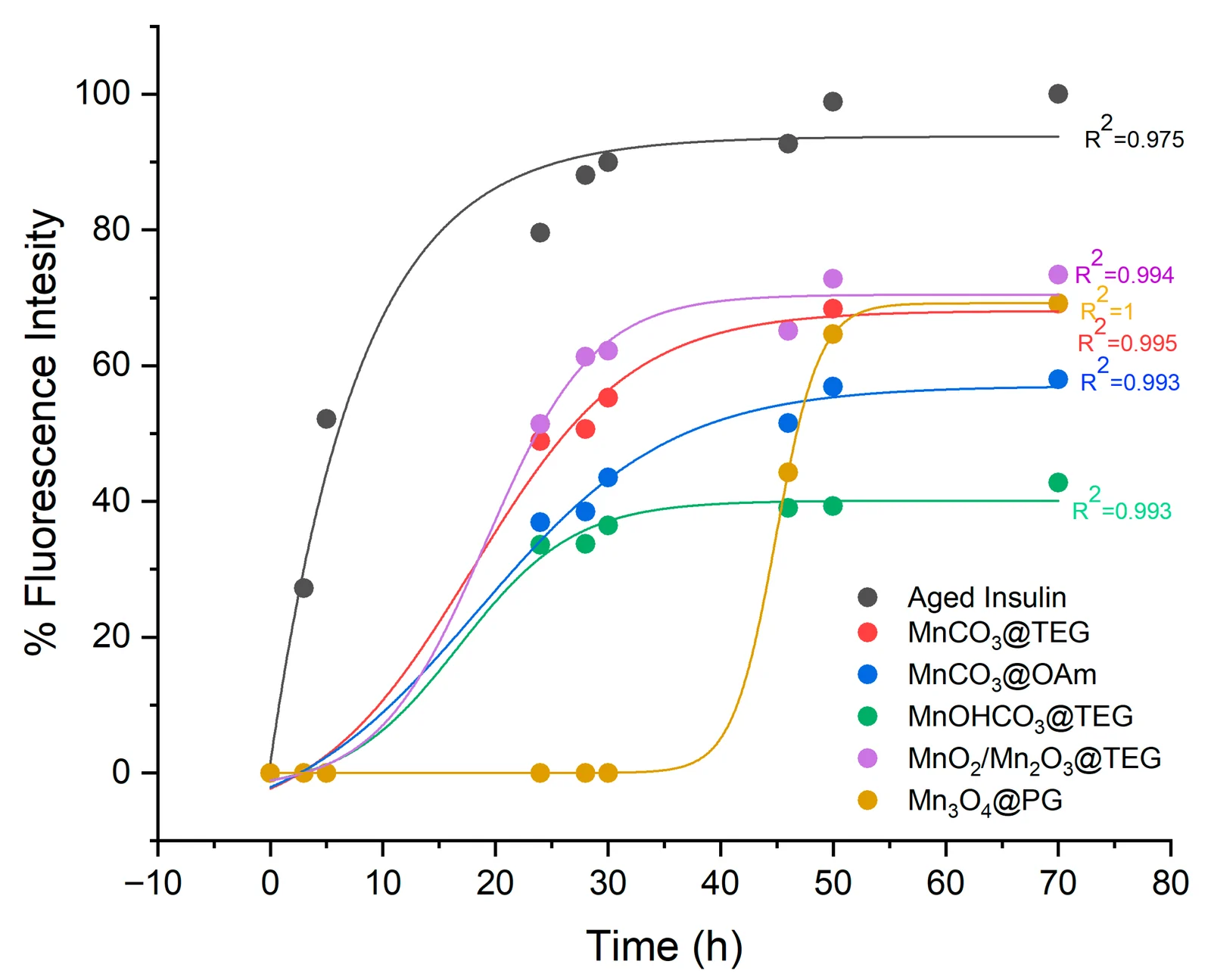
ThT fluorescence profiles of the aged insulin glargine (Lantus) formulation in the absence and presence of manganese-based nanoparticles.

The capacity of the as-prepared manganese-based nanoparticles to modulate the polymerization and depolymerization kinetics of aged insulin glargine was systematically evaluated in order to investigate the effect of pre-formed oligomeric seeds on amyloidosis kinetics [37]. The kinetics of aged insulin glargine in the absence/presence of manganese-based nanoparticles are given in Figure 5. In the absence of nanoparticles, fluorescence intensity increases already from the first 3 h (aggregation percentage = 27.19%) with subsequent rapid stabilization, indicating equilibrium and rapid protein polymerization, which is consistent with pre-existing ThT-positive aggregates and the absence of a detectable lag phase. In contrast, in the five cases where incubation was performed in the presence of nanoparticles, a delay in the phenomenon was observed, with the fluorescence signal showing an increase for most of the solutions after 24 h. The kinetic parameters of aged insulin amyloidosis are given in Table 5. In the control sample, the pre-existing ThT-positive aggregates were associated with an immediate rise in fluorescence, evidenced by a $t_{lag}$ and $t_{1/2}$ of 0 h, alongside an apparent elongation rate constant ($k_{app}$) of 0.124 h^−1^. However, all nanoparticle systems re-established an apparent lag phase, delaying the rise in ThT fluorescence. The Mn_3_O_4_@PG NPs caused the most profound kinetic delay, extending the $t_{lag}$ to 41.03 h and the $t_{1/2}$ to 44.89 h, although this system subsequently exhibited the fastest elongation rate ($k_{app}=0.518$ h^−1^) once fibrillation began. The remaining nanosystems demonstrated moderate temporal delays, with $t_{lag}$ values ranging from 1.26 h for MnCO_3_@OAm NPs up to 9.49 h for MnO_2_/Mn_2_O_3_@TEG NPs and corresponding $k_{app}$ values varying between 0.116 and 0.207 h^−1^. The MnOHCO_3_@TEG NPs produced the lowest terminal relative ThT signal (42.78%). A noticeable reduction was also achieved by the MnCO_3_@OAm NPs, which produced a terminal relative ThT signal of 58%. The MnCO_3_@TEG and Mn_3_O_4_@PG NPs produced comparable terminal relative ThT signals of 69.14% and 69.29%, respectively. Finally, the MnO_2_/Mn_2_O_3_@TEG NPs, despite being the least effective among the series, produced a terminal relative ThT signal of 73.40%.

**Figure 6:**
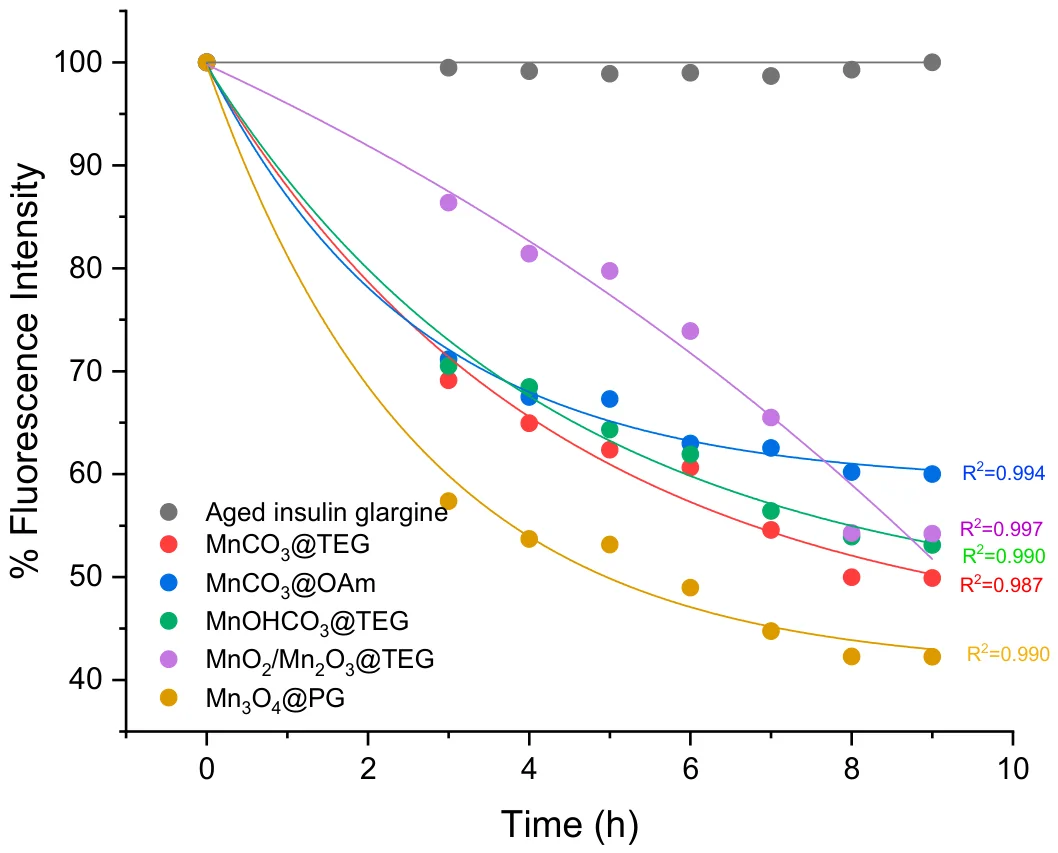
Kinetics of aged insulin glargine depolymerization in the absence/presence of manganese-based nanoparticles.

As far as the ThT decay assay is concerned, the fluorescence decay kinetics of aged insulin glargine aggregates in the absence/presence of Mn-based NPs are given in Figure 6, while the kinetic parameters were calculated based on a one-phase exponential decay function with a time constant parameter (Equation (5)), and they are given in Table 6. In the aged insulin glargine control, fluorescence remains constant (~100%), indicating no spontaneous decrease in ThT fluorescence and serving as the baseline for comparison. The MnCO_3_@OAm nanoparticles (blue) show the smallest reduction compared to neat insulin. After that, they follow the MnO_2_/Mn_2_O_3_@TEG NPs, which exhibit a sharp decrease of 54.2%. The MnOHCO_3_@TEG (green) and MnCO_3_@TEG (red) NPs display quite similar behavior (53.11% and 49.9%, respectively). Finally, the Mn_3_O_4_ NPs (yellow) produced the largest decrease in ThT fluorescence (42.26% remaining signal). These NPs produced the largest ThT signal decrease among the tested formulations.

The fluorescence of the aged insulin glargine control remains constant (~100%), indicating no spontaneous decrease in ThT fluorescence and serving as the baseline for comparison. The MnCO_3_@OAm nanoparticles (blue) show the smallest reduction compared to neat insulin. After that, they follow the MnO_2_/Mn_2_O_3_@TEG NPs, which exhibit a sharp decrease of 54.2%. The MnOHCO_3_@TEG (green) and MnCO_3_@TEG (red) NPs display quite similar behavior (53.11% and 49.9%, respectively). Finally, the Mn_3_O_4_ NPs (yellow) produced the largest decrease in ThT fluorescence (42.26% remaining signal). The kinetic profiles demonstrate that all manganese-based nanostructures produce a time-dependent decrease in amyloid-associated ThT fluorescence from aged insulin glargine aggregates, while the insulin fibril control (black) remains essentially constant at 100% over the entire 9 h incubation period (Figure 6). The Mn_3_O_4_ nanoparticles (yellow) exhibit the most pronounced effect, causing a rapid reduction in fluorescence down to approximately 40%, representing the largest fluorescence decrease under the examined conditions. MnCO_3_@TEG (red), MnCO_3_@OAm (blue), MnOHCO_3_@TEG (green), and MnO_2_/Mn_2_O_3_@TEG (purple) also induce a marked decrease in fluorescence, reaching final values around 50–60%, representing smaller fluorescence decreases than that produced by Mn_3_O_4_. The decay curves display an exponential-like behavior, empirically described by a first-order or pseudo-first-order fluorescence decay model; this fit does not establish structural disassembly. The high coefficients of determination (R^2^ ≈ 0.98–0.99) obtained from the kinetic fits further support the reliability of the applied model and allow a comparison of the apparent ThT decay rates among the different Mn-based nanomaterials.

The aged insulin glargine control shows no measurable kinetic constants and retains 100% relative ThT signal, indicating that no spontaneous decrease in the ThT signal was detected under these conditions. In contrast, all nanoparticle formulations decrease both the apparent half-life and the terminal relative ThT signal, with Mn_3_O_4_ NPs exhibiting a very short $\;t_{1/2}$ (1.40 h), a high apparent rate constant $k_{\mathrm{app}}$ (0.49 h^−1^), and the lowest terminal relative ThT signal (42.26%), representing the largest ThT signal decrease in the series. MnCO_3_@OAm NPs also display the fastest kinetics ($t_{1/2}=1.54$ h, $k_{\mathrm{app}}=0.44$ h^−1^) but leave a higher relative ThT signal (60%), whereas MnCO_3_@TEG and MnOHCO_3_@TEG NPs act more moderately, with longer half-lives (2.99 h and 2.95 h, respectively) and terminal relative ThT signals around 50–53%.

### 2.3. In Vitro Antioxidant Activity of Manganese-Based NPs

The antioxidant activity of the nanoparticles was investigated using a hydrogen peroxide (H_2_O_2_) scavenging assay. This assay is a standard in vitro spectrophotometric method utilized to evaluate the direct antioxidant capacity of chemical compounds and nanomaterials. Hydrogen peroxide is a naturally occurring, reactive non-radical species. While H_2_O_2_ itself is not highly reactive, it can easily penetrate cell membranes and undergo transition metal-catalyzed reactions (such as the Fenton reaction) to generate the highly toxic hydroxyl radical, which causes severe oxidative damage to lipids, DNA, and proteins. Manganese nanoparticles exhibit a catalase-like (CAT-like) multi-enzymatic activity, facilitating the degradation of H_2_O_2_ [38]. H_2_O_2_ exhibits a characteristic absorption maximum at approximately 230 nm. When the nanoparticles are introduced to the solution, their scavenging activity leads to the degradation of H_2_O_2_, which corresponds to a proportional decrease in the UV absorbance at 230 nm of the material’s antioxidant effectiveness.

**Figure 7:**
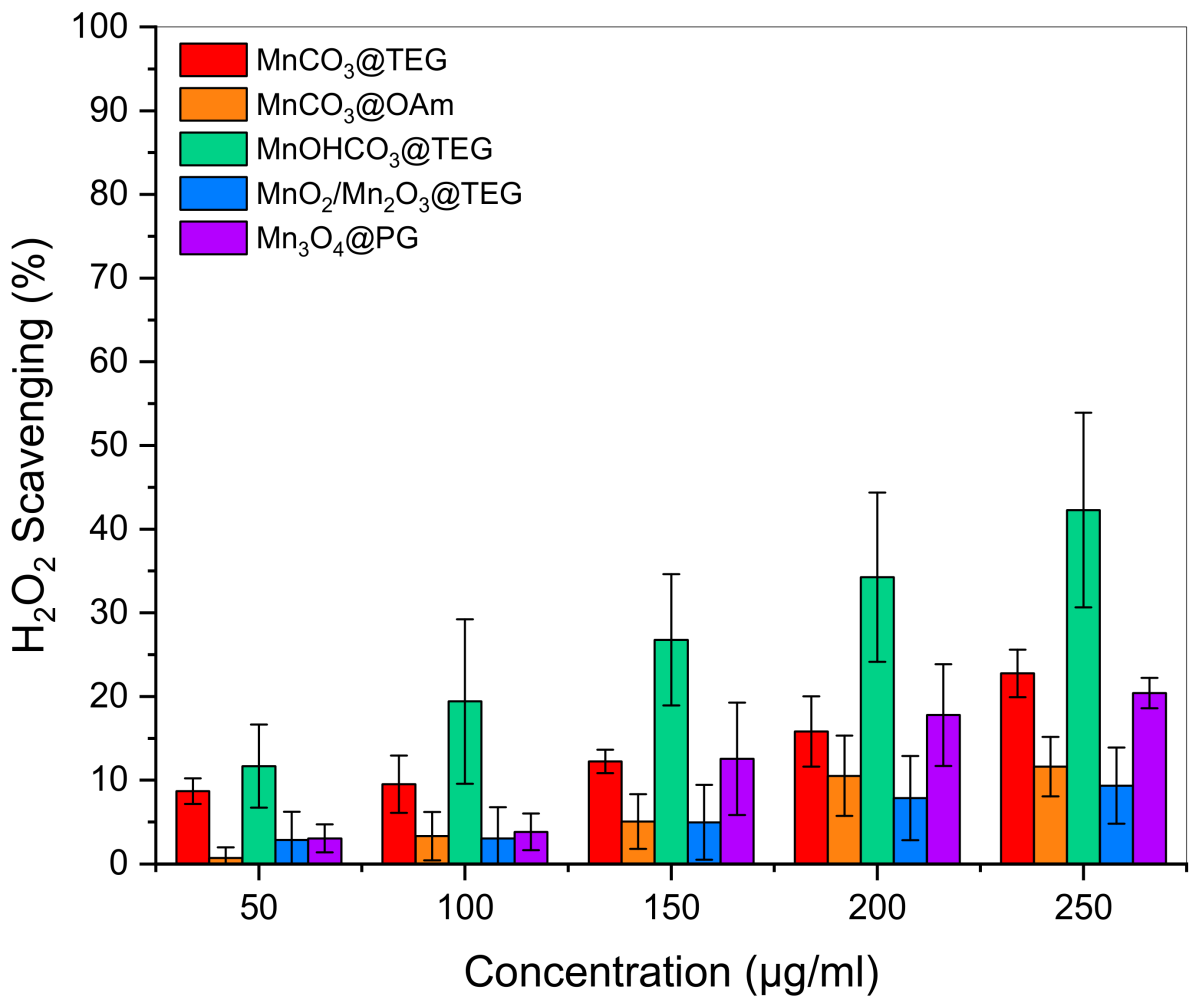
Bar chart illustrates the antioxidant activity of manganese-based NPs across different concentrations. Bars show mean ± SD from three independent measurements (*n* = 3).

The results from the antioxidant assay are given in Figure 7, while the respective IC_50_ values are in Table 7. MnOHCO_3_@TEG is the most efficient H_2_O_2_ scavenger. Its activity increased from 11.69% at 50 μg/mL to 42.27% at 250 μg/mL, and it was the only sample with a measurable IC_50_ value (306 μg/mL), indicating strong catalytic activity. Both MnCO_3_@TEG and Mn_3_O_4_@PG had moderate, dose-dependent antioxidant effects. MnCO_3_@TEG reached a maximum scavenging efficiency of 22.76% at 250 μg/mL, while Mn_3_O_4_@PG reached 20.42%. Although neither reached the IC_50_ threshold (>400 μg/mL), they maintain a steady, continuous catalytic interaction with the reactive oxygen species. However, MnCO_3_@OAm and MnO_2_/Mn_2_O_3_@TEG exhibited very poor antioxidant activity, peaking at 11.63% and 9.35%, respectively, at the highest tested concentration (250 μg/mL). Their IC_50_ values remain far above the >400 μg/mL threshold, rendering them virtually ineffective as standalone radical scavengers.

## 3. Discussion

In this study, five samples of functionalized manganese-based nanoparticles, MnCO_3_@TEG, MnCO_3_@OAm, MnOHCO_3_@TEG, MnO_2_/Mn_2_O_3_@TEG, and Mn_3_O_4_@PG, have been prepared using the solvothermal method based also on our previous results [39,40]. The NPs have been functionalized with polyols, which can act as a toolbox, since they offer control over particle size, morphology, and surface chemistry [41,42]. The nature of the polyol may affect polarity, viscosity, boiling point, coordinating ability of the system, and, therefore, affect how the precursor dissolves and how fast particle growth occurs. The heating profile controls the balance between nucleation and growth and influences how crystalline the particles become. The concentration and chemical form of the precursor determine the level of supersaturation and whether intermediate complexes are formed. Furthermore, adding base affects deprotonation of the polyol, the intermediate phases, and the kinetics of the reduction [43]. The physicochemical characterization of the Mn-based NPs confirmed the successful synthesis of the different functionalized well-defined nanostructures, which can be further exploited in biological studies.

For the fresh insulin aspart formulation, all nanoparticle-containing samples altered the fitted ThT profiles. MnCO_3_@TEG produced the longest apparent lag time (105.41 h versus 21.16 h for the control), while MnOHCO_3_@TEG produced the lowest terminal signal. DLS size and zeta potential differed across formulations, but the present data establish associations only. Adsorption, steric effects, and electrostatic interactions are plausible hypotheses from the broader literature; they were not directly measured here [44,45,46,47].

In the depolymerization experiments on fresh insulin aspart aggregates, all Mn-based formulations reduced ThT fluorescence intensity, indicating a decrease in amyloid-associated ThT-positive species under the assay conditions. In the absence of nanoparticles, the ThT signal of insulin remained stable over time, indicating no spontaneous decrease in the amyloid-associated fluorescence signal [48]. Upon adding MnCO_3_@TEG, the ThT signal declined steadily to a plateau markedly lower than the initial baseline, consistent with remodeling or disassembly of ThT-positive species, although structural disassembly was not directly established. MnCO_3_@OAm produced a comparable decay profile with slightly faster kinetics, which may reflect hydrophobic interactions between its surface layer and exposed non-polar regions of the aggregate [49]. MnOHCO_3_@TEG NPs also reduced the ThT signal; however, a higher terminal signal remained than for the other systems. One possible interpretation is that its negative surface charge affects aggregate remodeling or ThT binding site accessibility. In contrast, MnO_2_/Mn_2_O_3_@TEG forms the largest, most aggregated colloidal clusters and produces a smaller fluorescence decrease; one possible explanation is that aggregation-related steric effects limit access to the amyloid matrix [48,49]. Finally, Mn_3_O_4_@PG nanoparticles, combining small hydrodynamic size, high colloidal stability, and strong surface charge, produced the largest decrease in ThT fluorescence, reaching the lowest terminal signal.

When evaluated against the aged insulin glargine system, the nanoparticles produced distinct changes in the amyloid-associated ThT profiles. In the control group, aged insulin glargine aggregated almost instantaneously without an observable lag phase, consistent with pre-existing ThT-positive aggregates and the absence of a detectable primary nucleation phase [45,50]. Introducing MnCO_3_@TEG, MnCO_3_@OAm, MnOHCO_3_@TEG, and Mn_3_O_4_@PG NPs re-established a distinct lag phase while reducing the final ThT signal intensity. This re-emergence of an apparent lag phase may be consistent with capping of elongation sites or sequestration of secondary nucleation sites [51]. MnCO_3_@OAm exhibited similar inhibitory trends with extended characteristic times, which may reflect partial blocking of elongation sites by its hydrophobic shell. MnOHCO_3_@TEG, which had the most negative surface charge and was well dispersed, increased the apparent lag time and lowered the ThT signal; this may indicate fewer accessible ThT-binding sites, although the structural state of insulin was not determined. In contrast, MnO_2_/Mn_2_O_3_@TEG, which formed larger and more aggregated particles, mainly slowed the kinetics but retained a high terminal ThT signal, possibly because its clustered state limits interactions with the aggregates. The small, spinel-structured Mn_3_O_4_@PG nanoparticles induced the largest kinetic shift, generating a prolonged lag phase and the lowest plateau intensity.

During the ThT decay assays on aged insulin glargine, all Mn-based systems decreased the amyloid-associated fluorescence signal, whereas the signal remained stable in the control. MnCO_3_@TEG NPs decreased ThT intensity with a characteristic half-time of a few hours, a change consistent with remodeling or disassembly of ThT-positive species; structural disassembly was not directly established. MnCO_3_@OAm yielded a similar exponential decay but left approximately 60% of the initial ThT signal; its hydrophobic surface profile may contribute to this behavior. MnOHCO_3_@TEG NPs produced a rapid ThT-signal decrease comparable to MnCO3@TEG NPs. However, MnO_2_/Mn_2_O_3_@TEG left over half of the initial ThT signal intact; its large hydrodynamic diameter (624 nm) may limit access to dense aggregate networks [48,49]. Mn_3_O_4_@PG NPs produced the largest response in this assay, rapidly reducing the ThT signal to roughly 40% of its initial value, suggesting an association between small particle size, high colloidal dispersity, and a larger ThT signal decrease; structural confirmation is still required.

Beyond amyloid modulation, catalytic evaluation revealed that the synthesized nanoparticles possess intrinsic catalase-like H_2_O_2_ scavenging ability. MnOHCO_3_@TEG nanoparticles exhibited the highest scavenging activity (42.27% inhibition at 250 µg/mL), nearly doubling the performance of pure MnCO_3_@TEG NPs (22.76%). This enhanced performance may be related to structural hydroxyl (-OH) groups in the basic manganese carbonate lattice, which could increase surface hydrophilicity and promote substrate adsorption [52,53]. Furthermore, the high negative zeta potential of MnOHCO_3_@TEG NPs (−38.7 mV) may promote electrostatic repulsion and colloidal stability during incubation, thereby maintaining surface accessibility for Mn^2+^/Mn^3+^ redox cycling [54]. Conversely, near-neutral or low-charge formulations, such as MnO_2_/Mn_2_O_3_@TEG (−4.1 mV) and MnCO_3_@OAm (−14.7 mV), suffered from rapid precipitation and severe agglomeration (9.35% and 11.63%, respectively) [55].

Overall, these findings highlight the potential of Mn-based nanoparticles as multifunctional platforms for modulating insulin amyloid aggregation while simultaneously providing antioxidant activity. The observed differences among the formulations suggest that nanoparticle size, surface charge, dispersion state, and composition may be important parameters influencing their interactions with insulin aggregates at different stages of fibril formation. Although these associations remain preliminary and require confirmation by complementary structural methods and biological safety studies, they provide a foundation for the rational design of Mn-based nanomaterials with optimized physicochemical properties. Future studies integrating direct structural characterization, mechanistic investigations, and comprehensive biocompatibility assessment will be essential to determine whether this dual anti-amyloid and antioxidant potential can be translated into therapeutically relevant strategies for insulin amyloidosis.

## 4. Materials and Methods

### 4.1. Materials

All the reagents utilized for our total conducted experiments were of analytical grade and were used without any further purification: potassium permanganate ΚMnO_4_ (Aldrich, ≥99.9%, MW: 158.03), manganese(II) chloride tetrahydrate MnCl_2_·4H_2_O (Merck (Rahway, NJ, USA) ≥ 99.9% MW: 197.9), manganese(II) acetylacetonate Mn(acac)_2_ (Aldrich ≥ 99.9%, MW: 253.15), manganese(II) nitrate hydrate Mn(NO_3_)_2_·xH_2_O (Aldrich ≥ 99,9%, MW: 178.87), manganese(III) acetylacetonate Mn(acac)_3_ (Aldrich ≥ 99.9%, MW: 352.26), propylene glycol (PG) C_3_H_8_O_2_ (Merck ≥ 99.9%, MW: 76.09), tetraethylene glycol (TEG) C_8_H_18_O_5_ (Merck ≥ 99.9%, MW: 194.23), sodium hydroxide NaOH (MW: 39.98), oleylamine (OAm) C_18_H_35_NH_2_ (J & K Scientific (San Jose, CA, USA) ≥ 70%, MW: 286.33), hydrogen peroxide 30 weight % solution (Chem-Lab (Zedelgem, Belgium), MW: 34.01 g/mol), and thioflavin T (ThT) (J&K Scientific, 98%). Two commercial insulin analogues were used: NovoRapid (insulin aspart; Novo Nordisk (Bagsværd, Denmark); 100 units/mL solution for injection) as the initially non-aged formulation and Lantus (insulin glargine; Sanofi (Paris, France); 100 units/mL solution for injection) as the experimentally aged formulation. Lantus (Paris, France) was aged at pH 2.0 and 60 degrees C with shaking at 600 rpm for 6 h. Phosphate-Buffered Saline (PBS) (VWR, pH = 7.4) and dimethyl sulfoxide (DMSO) (Sigma-Aldrich, St. Louis, MO, USA, M = 78.13 g/mol) were used.

### 4.2. Synthesis of Functionalized Manganese-Based Nanoparticles

**Synthesis of S1**: A total of 0.2 g of KMnO_4_ and 8 mL of tetraethylene glycol (TEG) were weighed and added to a beaker, and the mixture was stirred for 10 min on a magnetic stirrer. The precursor KMnO_4_ does not fully dissolve in the TEG polyol. The color of the supernatant is initially dark purple and turns dark brown after 10 min. The entire content of the beaker is then transferred into a Teflon vessel, which is sealed inside a stainless steel autoclave and placed in an oven. The temperature of the oven is increased at a constant rate for one hour until it reaches 200 °C and is then maintained at this value for 24 h. The autoclave is allowed to cool down to room temperature at a constant rate and is then opened. The reaction mixture is transferred into Falcon tubes, where it is washed with ethanol. The suspension is centrifuged at 5000 rpm for 20 min, the supernatant is decanted, and the solid precipitate is collected. This washing and centrifugation procedure is repeated three times, after which the product is transferred to a round-bottom flask, and the solvent is removed and the sample dried using a rotary evaporator under vacuum. The remaining solid is scraped off the walls of the flask as a powder, weighed, and stored in an Eppendorf tube.

**Synthesis of S2:** In a beaker, 0.2 g of MnCl_2_·4H_2_O and 2 mL of propylene glycol (PG) were combined and stirred for 5 min on a magnetic stirrer. In a separate beaker, 4 mL of oleylamine, acting as a reducing agent, and 2 mL of propylene glycol were added and stirred for 5 min on a magnetic stirrer. Following this, the contents of the second beaker (OAm, PG) are added to the first beaker and stirred for an additional 8 min on a magnetic stirrer. Upon the addition of the reducing agent, the supernatant immediately changes color and turns light brown. The mixture is then transferred into a Teflon vessel, sealed inside an autoclave, and placed in an oven. Afterwards, the same procedure described for the preparation of sample S1 was followed.

**Synthesis of S3:** In a beaker, 0.2 g Mn(acac)_2_ and 4 mL of tetraethylene glycol (TEG) were combined and stirred for 5 min on a magnetic stirrer. Mn(acac)_2_ does not fully dissolve in TEG, and the supernatant appears light brown. In a separate beaker, 0.2 g of NaOH, used as a reducing agent, and 4 mL of TEG were added and stirred for 5 min on a magnetic stirrer. The content of the second beaker (NaOH, TEG) was then added to the first beaker, and the resulting mixture was stirred for an additional 8 min on a magnetic stirrer. The mixture was subsequently transferred into a Teflon vessel, sealed inside an autoclave, and placed in an oven. The same procedure as described for the preparation of sample S1 was then followed.

**Synthesis of S4:** In a beaker, 0.2 g Mn(NO_3_)_2·_xH_2_O and 8 mL of tetraethylene glycol (TEG) were added and stirred for 10 min on a magnetic stirrer. Mn(NO_3_)_2_·xH_2_O is completely soluble in TEG, and the resulting solution is colorless and transparent. After 10 min of stirring, the contents of the beaker were transferred into a Teflon vessel, which was sealed inside an autoclave and placed in an oven. The same procedure as described for the preparation of sample S1 was then followed.

**Synthesis of S5:** In a beaker, 0.2 g Mn(acac)_3_ and 4 mL of propylene glycol (PG) were combined and stirred for 5 min on a magnetic stirrer. Mn(acac)_3_ does not fully dissolve in PG, and the supernatant is dark brown. In a separate beaker, 0.2 g of NaOH, used as a reducing agent, and 4 mL of PG were added and stirred for 5 min on a magnetic stirrer. The content of the second beaker (NaOH, PG) was then added to the first beaker, and the mixture was stirred for an additional 8 min on a magnetic stirrer. The mixture was then transferred into a Teflon vessel, sealed inside an autoclave, and placed in an oven. The same procedure as described for the preparation of sample S1 was then followed.

### 4.3. Characterization Methods

The crystal structure and crystallite size of synthesized NPs were investigated through X-ray diffraction (XRD) using a Siemens Diffraktometer D5000 performed in the 2*θ* region from 10 to 70°, with monochromatized Cu-Ka X-ray radiation (λ = 1.5418 Å) and a curved crystal graphite monochromator operating at 45 kV and 100 mA; counts were accumulated every 0.020 (2*θ*) with a counting time of 2 s per step. A Nicolet series FT-IR spectrometer (Nicolet iS20, Thermo Fisher Scientific, Waltham, MA, USA) with a monolithic diamond ATR crystal was used to acquire the ATR-FTIR spectra (4000−450 cm^−1^) of the NPs and were displayed as transmittance. TGA was utilized to calculate the amount of organic coating of NPs using SETA-RAM SetSys-1200 at a heating rate from 30 °C to 800 °C (10^o^ min^−1^) under argon. The antioxidant properties were evaluated using a UV-Vis spectrophotometer (V-750, Jasco, Tokyo, Japan). The mean diameter (d.nm), polydispersity index (PDI), and the zeta potential of the NPs were determined by dynamic light scattering (DLS) on a Zetasizer Nano ZS (Malvern Panalytical, Malvern, UK).

### 4.4. ThT Studies of Fresh Insulin Aspart and Aged Insulin Glargine Formulations

Insulin samples were diluted to 1 mg/mL for the ThT assays. The fresh system used NovoRapid (insulin aspart), whereas the aged system used Lantus (insulin glargine) that had been aged at pH 2.0 and 60 degrees C with shaking at 600 rpm for 6 h to generate a preparation containing pre-existing ThT-positive aggregates. Subsequent assay samples were incubated at approximately 37 degrees C with agitation at 130 rpm. Nanoparticles were added at a protein-to-nanoparticle mass ratio of 10:1 (100 micrograms/mL nanoparticle) while maintaining a constant protein concentration. ThT stock solution (3.1 mM in PBS) was filtered through a 0.2-micrometer syringe filter and protected from light. At each time point, 20 microliters of insulin sample was diluted into 2 mL PBS containing 10 microliters of ThT stock (approximately 15 micromolar final ThT). Fluorescence emission was recorded from 460 to 600 nm using a Hitachi F-7000 spectrofluorometer; the signal near 485 nm was used for analysis, with 5 nm excitation and emission slit widths. Signals were normalized separately to the formulation-matched control. The operational percentage aggregation was calculated as 100× (terminal ThT signal of the nanoparticle-containing sample/terminal ThT signal of the corresponding insulin control). This quantity is a relative fluorescence endpoint and not a direct measurement of fibril mass. Nanoparticle-only controls containing ThT and each nanoparticle formulation, but no insulin, were evaluated at the same nanoparticle concentration and under the same fluorescence settings; no detectable signal was observed in the monitored ThT emission range. This control excludes intrinsic nanoparticle fluorescence but does not exclude scattering, quenching, displacement of fibril-bound ThT, or altered dye binding site accessibility in the complete assay. Boltzmann’s sigmoidal Equation (1) was used to fit the aggregation-associated fluorescence profiles:

(1) $$Y\left(t\right)={Ι}_{2}+\;\frac{{Ι}_{1}-\;{Ι}_{2}}{1+\;e^{\frac{-\left(t-t_{1/2}\right)}{\tau }}}\;$$

where *Y*(*t*) represents the percentage of fibrillated protein as a function of incubation time, *t*; *I*_1_ and *I*_2_ are ThT fluorescence intensities of initial insulin monomers and final amyloids, respectively; *t*_1/2_ stands for the time required for the 50% completion of amyloid formation (growth halftime); and *τ* represents the time constant. In this way, it is possible to evaluate the time course of the process and extract the kinetic parameters, such as elongation rate constant (*k_app_*) and lag time (*t_lag_*). Lag time was approximated as follows:

(2) $$t_{lag}=\;t_{1/2}-2\tau$$

whereas the elongation rate was given by Equation (3):

(3) $$k_{app}=\frac{1}{\tau }$$

**Study of ThT fluorescence decay kinetics in insulin aggregate samples:** The fluorescence decay profiles of fresh insulin aspart aggregates were modeled using a third-degree polynomial (cubic) function to ensure the highest quality of fit across the complex fluorescence decay profiles:

(4) $$y=A+Bx+Cx^{2}+Dx^{3}$$

where y represents the fluorescence intensity (%) and x represents the incubation time (h). From these high-fidelity fits (R^2^ > 0.99), the apparent half-life ($t_{1/2}$) and the apparent rate constant ($k_{app}$) were extracted to allow a meaningful comparison among the different manganese-based nanomaterials. The fluorescence decay profiles of aged insulin aggregate samples were better described by a one-phase exponential decay function with a time constant parameter:

(5) $$y=A1\;\times exp\left(-x,/,t_{1}\right)+y_{0}$$

where t_1_ is the decay time constant (in hours), the rate constant is $k=\;1/t_{1}$, and the half-life is $t_{1/2}=\;t_{1\;}\times ln\left(2\right)\;\approx 0.693\;\times \;t_{1}$.

### 4.5. In Vitro Antioxidant Activity of Mn-Based NPs by H_2_O_2_ Assay

To evaluate the scavenging capacity of the manganese-based nanoparticles, a standard H_2_O_2_ degradation assay was performed, with optical density measurements recorded at λ_max_ = 230 nm. The experimental design required a final H_2_O_2_ concentration of 10 mM within the measurement cuvette. To test the dose-dependent antioxidant activity, varying concentrations of the nanoparticle (50, 100, 150, 200, and 250 μg/mL) were tested. The addition of hydrogen peroxide initiated the reaction, and the samples were incubated for 20 min at a temperature of approximately 18 °C. To prevent any photo-induced degradation of the reagents, the entire experimental procedure was conducted in the dark. The percentage of hydrogen peroxide scavenging capacity was calculated using the following equation:

(6) $$\%\;Scavenging=\left[\frac{A_{ControlA}-\left(A_{Sample}-A_{ControlB}\right)}{A_{ControlA}}\right]\times 100$$

IC_50_ values were estimated from concentration–response curve fitting by determining the fitted concentration corresponding to 50% scavenging.

### 4.6. Statistical Analysis

OriginPro 2018 was used for curve fitting and descriptive analysis. Values are reported as mean ± standard deviation from three independent measurements (n = 3); the error bars in Figure 7 represent standard deviation. No inferential hypothesis tests or multiplicity corrections were performed. Fitted lag times, half times, rate parameters, and terminal fluorescence percentages are, therefore, interpreted descriptively.

## Figures and Tables

**Table 2:** Dispersion parameters of manganese-based nanoparticles. PDI is reported as a dimensionless value.

Sample	Z-Average Hydrodynamic Diameter (nm)	PDI	Zeta (mV)
MnCO_3_@TEG	226	0.183	−24.3
MnCO_3_@OAm	324	0.299	−14.7
MnOHCO_3_@TEG	196	0.278	−38.7
MnO_2_/Mn_2_O_3_@TEG	624	0.41	−4.1
Mn_3_O_4_@PG	101	0.13	−36.93

**Table 3:** Fitted ThT profile parameters and relative terminal ThT signal for the fresh insulin aspart formulation in the absence and presence of manganese-based nanoparticles.

	$t_{lag}$ (h)	$t_{1/2}$ (h)	$k_{app}$ (h^−1^)	%Aggregation
Fresh insulin aspart (NovoRapid)	21.16	45.96	0.081	100
MnCO_3_@TEG NPs	105.41	123.24	0.112	85.8
MnCO_3_@OAm NPs	41.35	47.40	0.331	79.08
MnOHCO_3_@TEG NPs	60.42	69.78	0.214	69.61
MnO_2_/Mn_2_O_3_@TEG NPs	36.49	54.20	0.113	89.71
Mn_3_O_4_ @PG NPs	63.95	73.81	0.203	73.91

**Table 4:** Summary table of the calculated depolymerization kinetic parameter half-lives $t_{1/2}$, *k_app_*, and % aggregation values of fresh insulin aspart in the presence/absence of Mn-based NPs.

	$t_{1/2}$ (h)	$k_{app}$ (h^−1^)	%Aggregation
Fresh insulin aspart (NovoRapid)	-	-	100
MnCO_3_@TEG NPs	3.27	0.211	59
MnCO_3_@OAm NPs	2.16	0.32	57
MnOHCO_3_@TEG NPs	2.85	0.243	65
MnO_2_/Mn_2_O_3_@TEG NPs	3.32	0.208	70
Mn_3_O_4_ @PG NPs	3.23	0.214	53

**Table 5:** Fitted ThT profile parameters and relative terminal ThT signal for the aged insulin glargine formulation in the absence and presence of manganese-based nanoparticles.

	$t_{lag}$ (h)	$t_{1/2}$ (h)	$k_{app}$ (h^−1^)	%Aggregation
Aged insulin glargine (Lantus)	0	0	0.124	100
MnCO_3_@TEG NPs	3.83	17.97	0.141	69.14
MnCO_3_@OAm NPs	1.26	18.48	0.116	58
MnOHCO_3_@TEG NPs	6.53	16.77	0.195	42.78
MnO_2_/Mn_2_O_3_@TEG NPs	9.49	19.13	0.207	73.40
Mn_3_O_4_@PG NPs	41.03	44.89	0.518	69.29

**Table 6:** Summary table of calculated kinetic parameters $t_{1/2}$, *k_app_*, and % aggregation values of aged insulin glargine in the presence/absence of Mn-based NPs.

	$t_{1/2}$ (h)	$k_{app}$ (h^−1^)	%Aggregation
Aged Insulin Glargine(Lantus)	-	-	100
MnCO_3_@TEG NPs	2.99	0.231	49.9
MnCO_3_@OAm NPs	1.81	0.381	60
MnOHCO_3_@TEG NPs	2.95	0.234	53.11
MnO_2_/Mn_2_O_3_@TEG NPs	-	-	54.2
Mn_3_O_4_@PG NPs	1.82	0.379	42.26

**Table 7:** H_2_O_2_ scavenging activity and IC_50_ of manganese-based nanoparticles at various concentrations.

Sample	50 μg/mL	100 μg/mL	150 μg/mL	200 μg/mL	250 μg/mL	Fit-Estimated IC_50_ μg/mL
MnCO_3_@TEG	8.71	9.52	12.25	15.83	22.76	>400
MnCO_3_@OAm	0.72	3.32	5.07	10.52	11.63	>400
MnOHCO_3_@TEG	11.69	19.41	26.78	34.26	42.27	306
MnO_2_/Mn_2_O_3_@TEG	2.86	3.06	4.97	7.85	9.35	>400
Mn_3_O_4_@PG	3.06	3.82	12.57	17.78	20.42	>400

## Data Availability

The original contributions presented in this study are included in the article. Further inquiries can be directed to the corresponding author(s).

## Abbreviations

The following abbreviations are used in this manuscript:

DLS	Dynamic Light Scattering
DMSO	Dimethyl Sulfoxide
FTIR	Fourier-Transform Infrared Spectroscopy
H_2_O_2_	Hydrogen Peroxide
MNPs	Manganese Nanoparticles
NPs	Nanoparticles
OAm	Oleylamine
PBS	Phosphate-Buffered Saline
PDI	Polydispersity Index
PG	Propylene Glycol
ROS	Reactive Oxygen Species
TEG	Tetraethylene Glycol
TGA	Thermogravimetric Analysis
ThT	Thioflavin-T
UV-Vis	Ultraviolet–Visible Spectroscopy
XRD	X-Ray Diffraction
